# Establishment of Reference Interval for CA72‐4 in Healthy Adults in Shenzhen, China

**DOI:** 10.1002/jcla.70188

**Published:** 2026-03-08

**Authors:** Mengting An, Zhiling Xu, Yanan Zhang, Junjie Wan

**Affiliations:** ^1^ Cancer Hospital & Shenzhen Hospital, Chinese Academy of Medical Sciences and Peking Union Medical College Shenzhen China

**Keywords:** CA72‐4, health check‐up, reference interval, regional difference, tumor marker

## Abstract

**Background:**

This study aimed to establish a reference interval (RI) for the tumor marker carbohydrate antigen 72‐4 (CA72‐4) in healthy adults in Shenzhen, China, and to validate its applicability.

**Methods:**

Serum CA72‐4 results from 2614 adults undergoing routine check‐ups were analyzed per CLSI EP28‐A3c. High outliers were removed with Tukey's 1.5 × IQR rule. Distribution normality was tested by Shapiro–Wilk, and the need for partitioning by sex or age was assessed with the Harris–Boyd *Z*‐test. The RI was derived non‐parametrically from the 2.5th and 97.5th percentiles; a 90% confidence interval (CI) for the upper limit was obtained by 2000‐bootstrap resampling. Performance was evaluated in an independent set of 206 adults (Apr 2025) by the exceedance rate (< 5% acceptable).

**Results:**

CA72‐4 values were markedly right‐skewed. After excluding 258 outliers (9.9%), 2356 results remained. No significant sex‐ or age‐related differences were found (*Z* < 3); thus, a combined RI was adopted. The 2.5th percentile was 0.54 U/mL (90% CI, 0.52–0.55) and the 97.5th percentile 8.5 U/mL (90% CI, 8.29–8.90). The oldest subgroup (61–85 year) showed a modest increase, but it did not affect the overall RI. In validation, 4.85% (10/206) exceeded 8.5 U/mL, meeting the preset criterion.

**Conclusions:**

An upper reference limit of 8.5 U/mL for CA72‐4 in Shenzhen healthy adults—higher than the manufacturer's value—reduces false positives in health screening. Slight age‐related increases suggest cautious interpretation in the elderly. This study provides an important reference for region‐specific tumor marker intervals in Shenzhen and similar populations.

## Graphical abstract

We established a reference interval (RI) for serum CA72‐4 in healthy adults in Shenzhen, China, based on data from 2614 individuals. Using CLSI EP28‐A3c guidelines, we determined an upper reference limit of 8.5 U/mL, which significantly reduced false‐positive results compared to the manufacturer's cutoff. This study provides practical guidance for regional laboratories and supports the localization of tumor marker RIs in China.

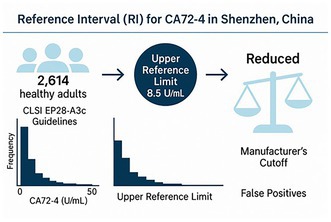

## 1 Introduction

CA72‐4 is a high‐molecular‐weight, tumor‐associated glycoprotein recognized by monoclonal antibodies B72.3 and CC49 against the TAG‐72 epitope [1]. Serum CA72‐4 rises markedly in several malignancies—especially gastric, gastrointestinal, and ovarian cancers. In gastric cancer, combining CA72‐4 with CEA and CA19‐9 enhances diagnostic sensitivity and aids postoperative monitoring [2, 3]. Because a single marker lacks adequate sensitivity and specificity for early screening, guidelines recommend CA72‐4 only as an adjunct for diagnosis and follow‐up rather than as an independent screen.

Reference intervals (RIs) for tumor markers are influenced by genetics, environment, diet, and assay methodology [4]. Substantial regional variation in CA72‐4 baselines has been documented: for instance, a Korean multicenter study showed assay‐dependent RI differences [5], and a Beijing cohort reported an upper limit of 18.0 U/mL in women aged 16–60 versus 14.5 U/mL in men, with no sex gap after 60 years. Such findings highlight the need for population‐specific RIs [ 6].

In our routine clinical practice, the health examination department has frequently observed that many asymptomatic individuals present with CA72‐4 levels exceeding the standard reference range provided by assay manufacturers. This phenomenon has led to unnecessary concern, psychological stress, and repeated consultations among examinees. These observations underscore the need to establish a locally appropriate reference interval that accurately reflects baseline CA72‐4 levels in the healthy Shenzhen population. Currently, no reference interval (RI) data for CA72‐4 exist for healthy adults in Shenzhen. To address this gap, we established and validated a local RI using a large health examination dataset and followed CLSI‐recommended procedures. Our findings provide a scientific basis for laboratories in Shenzhen and contribute to the regionalization of tumor marker reference intervals across China.

## 2 Materials and Methods

### 2.1 Study Population

This study included adults who underwent routine health check‐ups at the Medical Examination Center of the Cancer Hospital, Chinese Academy of Medical Sciences, Shenzhen, from January 2024 to March 2025. A total of 2614 healthy individuals (1565 men and 1049 women; age range 20–82 years, median 46 years) were identified through the hospital laboratory information system. All selected individuals had no recent history of malignant tumors, no significant organ diseases or acute infections detected in the check‐up, no gastrointestinal disorders, and (for women) were non‐pregnant. None had recent surgeries or were taking medications known to affect CA72‐4 levels. The “healthy” status was determined based on the absence of abnormal findings in the routine health check‐up package, which included a physical examination, abdominal ultrasound, chest radiography, and routine laboratory tests (liver and kidney function, blood glucose, lipids). Invasive procedures such as gastroscopy or CT scans were not performed on all participants unless clinically indicated. Additionally, in April 2025, a validation cohort of 206 healthy subjects meeting the same criteria was recruited from the check‐up center to evaluate the applicability of the established reference interval. This study was conducted in accordance with the *Guidelines for Exemption from Ethics Review for Medical and Health Institutions of Guangdong Province*. Article 2 of these Guidelines specifies that research relying exclusively on anonymized data is exempt from institutional ethical review. As the present work entailed only a retrospective analysis of fully de‐identified laboratory results obtained during routine health examinations, without additional sampling or intervention, it was deemed minimal‐risk and qualified for waiver of both ethical review and informed consent.

### 2.2 Assay Method

According to the standard operating procedure of the Medical Examination Center, all participants were instructed to fast for at least 8 h prior to blood collection. Samples showing visible lipemia or hemolysis were excluded during the initial laboratory screening. Serum was separated and the concentration of CA72‐4 was measured using a Roche Cobas e602 electrochemiluminescence immunoassay analyzer (Roche Diagnostics). The assay's analytical performance includes a total coefficient of variation (CV) of 2.3% and a lower detection limit of 0.5 U/mL. The laboratory participates in the National Center for Clinical Laboratories (NCCL) external quality assessment (EQA) scheme and strictly performs daily internal quality control (IQC) procedures. All measurements were conducted in the same laboratory under consistent conditions.

### 2.3 Statistical Analysis

Statistical processing adhered to CLSI EP28‐A3c [7]. Outliers were identified with Tukey's 1.5 × IQR rule—values ≥ Q_3_ + 1.5 IQR were excluded. Data normality was checked by the Shapiro–Wilk test; non‐normal distributions prompted use of a non‐parametric percentile method to derive the 95% reference interval (RI) from the 2.5th (P_2.5_) and 97.5th (P_97.5_) percentiles, with P_97.5_ taken as the upper reference limit. Precision of P_97.5_ was evaluated by a 2000‐resample bootstrap, yielding its 90% confidence interval. Potential partitioning by sex or age was assessed with the Harris–Boyd Z‐test (Z = |X̄_1_ − X̄_2_| / √[S_1_^2^/n_1_ + S_2_^2^/n_2_]); a *Z* ≥ 3 indicated a clinically meaningful difference requiring separate RIs. If no significant difference was found (*Z* < 3), a single combined reference interval was used for all groups. The final RI was validated in an independent cohort of 206 local healthy adults by calculating the exceedance rate—proportion of results outside the RI—with < 5% considered acceptable. Analyses were executed in Python 3.12.2.

## 3 Results

### 3.1 Baseline Distribution and Outlier Removal

**FIGURE 1:**
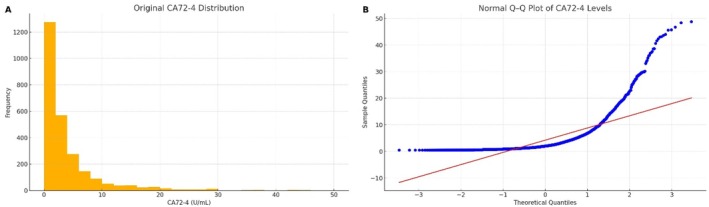
Distribution of serum CA72‐4 levels in the 2614 healthy adults (Shenzhen cohort) before outlier exclusion. (A) Histogram of CA72‐4 concentrations, showing a right‐skewed distribution. (B) Q–Q plot comparing the sample quantiles (vertical axis) with theoretical normal quantiles (horizontal axis), demonstrating deviation from normality.

Among the 2614 healthy adults, serum CA72‐4 concentrations exhibited a strongly right‐skewed distribution (Figure 1A). The majority of individuals (over 90%) had CA72‐4 levels below 5 U/mL, with only a small subset showing higher values, producing a long right‐side tail. Forty samples (1.5%) were below the assay's quantitation limit of 0.5 U/mL. The Q–Q plot of the data (Figure 1B) showed that the observed quantiles deviated substantially from the line of identity, especially at the high end, confirming a non‐normal distribution. Descriptive statistics for CA72‐4 stratified by sex and age group are presented in Table 1. Both males and females demonstrated a slight increase in median CA72‐4 with advancing age. For instance, among males the median value was 2.00 U/mL in the 18–40 year group versus 3.00 U/mL in the 61–85 year group; in females, the median was 2.14 U/mL in the youngest group versus 3.05 U/mL in the oldest group. The overall skewness and kurtosis were markedly above zero (Table 1), quantifying the right‐skewed and heavy‐tailed nature of the distribution.

**TABLE 1:** Distribution characteristics of serum CA72‐4 concentrations in the healthy check‐up population (before outlier removal).

Sex	Age group (years)	*N*	Median (U/mL)	SD	Skewness	Kurtosis
Overall	20–85 (all)	2614	2.05	5.94	3.46	18.13
Male	18–40	475	2.00	1.85	2.10	5.12
Male	41–60	951	2.50	2.07	2.30	6.01
Male	61–85	139	3.00	2.30	2.45	6.50
Female	18–40	411	2.14	1.92	2.20	5.30
Female	41–60	529	2.60	2.21	2.35	6.10
Female	61–85	109	3.05	2.40	2.50	6.60

*Note:* Skewness and kurtosis are Pearson moment coefficients of the sample distribution.

**FIGURE 2:**
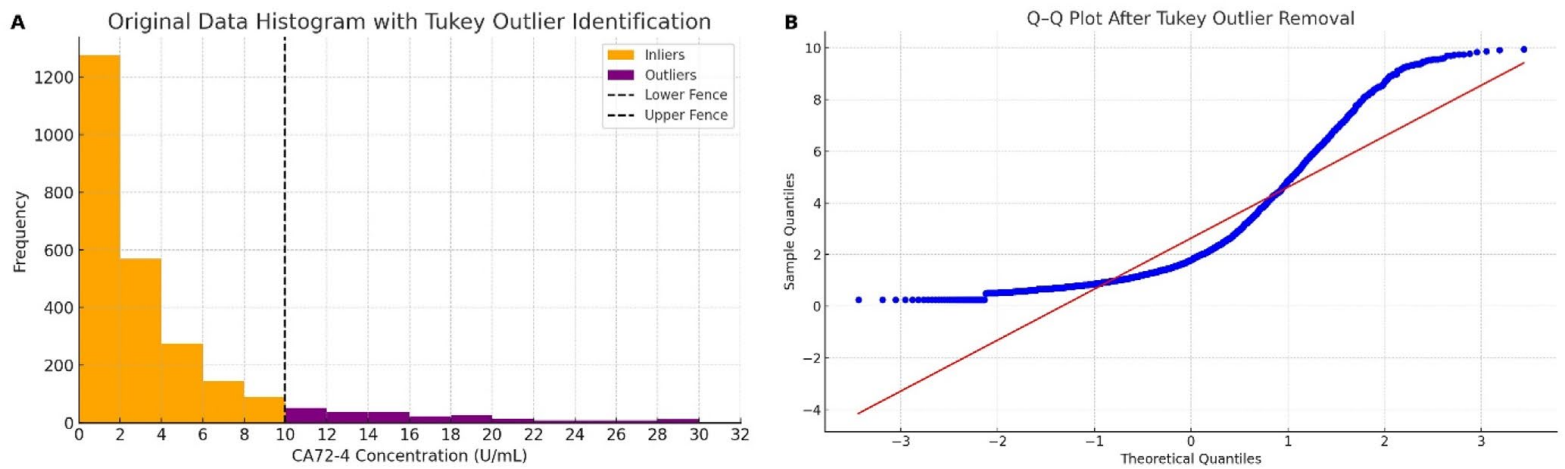
Analysis after exclusion of outliers by Tukey's method. (A) The shaded purple region indicates the samples identified as high outliers (CA72‐4 > 15 U/mL) that were removed prior to reference interval calculation. (B) Q–Q plot for the remaining 2356 samples after outlier removal, showing improved alignment but still some deviation from the normal reference line.

To reduce the impact of extreme values on reference interval calculation, we identified and removed outliers using Tukey's method. A total of 258 samples (approximately 9.9% of the dataset) were flagged as high‐end outliers (generally CA72‐4 > 15 U/mL, with a maximum observed value of 31.5 U/mL) and excluded from analysis (Figure 2A illustrates these outlier values in purple). We further analyzed the distribution of these 258 outliers and found no significant clustering in any specific age or sex subgroup, suggesting that the outlier exclusion process did not introduce systematic bias (data not shown). The Q–Q plot of the trimmed dataset (Figure 2B) continued to show departures from the normal reference line, though less extreme than before. After outlier removal, 2356 measurements remained, showing a more condensed distribution with a shorter tail (Table 2). Nonetheless, the distribution was still right‐skewed, and the Shapiro–Wilk normality test remained significant (*p* < 0.001), indicating that a nonparametric approach was appropriate.

**TABLE 2:** Distribution characteristics of serum CA72‐4 concentrations after removing outliers (Tukey's method).

Sex	Age group (years)	*N*	Median (U/mL)	SD	Skewness	Kurtosis
Overall	20–85 (all)	2356	1.80	2.16	1.41	4.27
Male	18–40	432	1.77	2.17	1.41	4.27
Male	41–60	862	1.78	2.15	1.50	4.47
Male	61–85	133	1.84	2.13	1.45	4.48
Female	18–40	371	1.68	2.13	1.48	4.66
Female	41–60	461	1.88	2.22	1.25	3.68
Female	61–85	97	2.31	1.92	1.15	3.94

*Note:* Skewness and kurtosis are Pearson moment coefficients of the sample distribution.

### 3.2 Reference Interval Calculation

**FIGURE 3:**
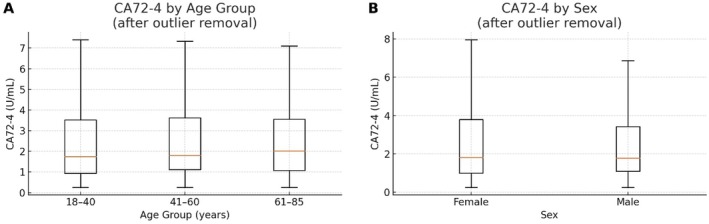
Box‐plot of serum CA72‐4 concentrations stratified by age (18–40, 41–60, 61–85 years) and sex group in the healthy Shenzhen population. The median values show a slight increasing trend in the oldest age group for both males and females, but the overlap between groups is considerable (no statistically significant differences by Harris–Boyd analysis).

We next evaluated whether separate reference intervals were needed for subgroups by sex or age. The Harris‐Boyd *Z*‐test comparisons (Table 3) revealed that none of the between‐group differences approached the threshold *Z*‐value of 3 for significance. In other words, CA72‐4 distributions did not differ significantly between males and females, nor between the younger (18–40 years), middle‐aged (41–60 years), and older (61–85 years) adult subgroups in this cohort. These results indicated that partitioning by sex or age was not necessary. The oldest age group did show a modest trend toward higher CA72‐4 values (higher medians), but this trend was not statistically significant and did not substantially affect the combined reference interval (Figure 3). Therefore, we pooled all 2356 remaining samples to establish a single reference interval for CA72‐4.

**TABLE 3:** Harris–Boyd *Z*‐test comparing CA72‐4 distributions by sex and age group.

Comparison group	*Z* value	Significant (Z ≥ 3)
Male 18–40 vs. Male 41–60	0.22	No
Male 41–60 vs. Male 61–85	0.35	No
Female 18–40 vs. Female 41–60	0.18	No
Female 41–60 vs. Female 61–85	0.40	No
Male 18–40 vs. Female 18–40	0.07	No
Male 41–60 vs. Female 41–60	0.07	No
Male 61–85 vs. Female 61–85	0.02	No

*Note:* A *Z*‐value ≥ 3 indicates a statistically significant difference.

Using the non‐parametric percentile method on the combined dataset, we found that the reference interval for serum CA72‐4 in Shenzhen healthy adults is 0.54 U/mL to 8.5 U/mL. The 2.5th percentile (lower limit) was approximately 0.54 U/mL, and the 97.5th percentile (upper limit) was 8.5 U/mL. The 90% confidence interval for the 97.5th percentile was 8.29–8.90 U/mL based on bootstrap resampling. Accordingly, the recommended upper reference limit for CA72‐4 in this population is 8.5 U/mL (expressed for reporting as “≤ 8.5 U/mL”). Table 4 summarizes the reference interval findings, including separate results for subgroups. Notably, when males and females were considered separately, their 97.5th percentiles were essentially the same (both 8.5 U/mL), reinforcing that a combined reference range is appropriate.

**TABLE 4:** Serum CA72‐4 reference intervals in Shenzhen healthy adults (nonparametric method).

Category	*N*	Median (U/mL)	P_2.5_ (90% CI)	P_97.5_ (90% CI)
Overall (Combined)	2356	1.80	0.54 [0.519, 0.550]	8.50 [8.29, 8.90]
Male	1427	1.79	0.55 [0.530, 0.586]	8.50 [8.241, 8.984]
Female	929	1.81	0.51 [0.250, 0.540]	8.50 [8.078, 8.884]
Age 18–40	803	1.74	0.25 [0.250, 0.511]	8.50 [8.150, 8.980]
Age 41–60	1323	1.80	0.59 [0.550, 0.620]	8.60 [8.376, 8.939]
Age 61–85	230	2.02	0.59 [0.535, 0.665]	7.80 [6.894, 9.386]

*Note:* “Overall” refers to the combined data of Shenzhen healthy check‐up subjects after outlier removal. The reference interval is defined as P_2.5_–P_97.5_ (0.54–8.5 U/mL), and the upper reference limit is reported as ≤ 8.5 U/mL.

### 3.3 Validation of the Reference Interval

**FIGURE 4:**
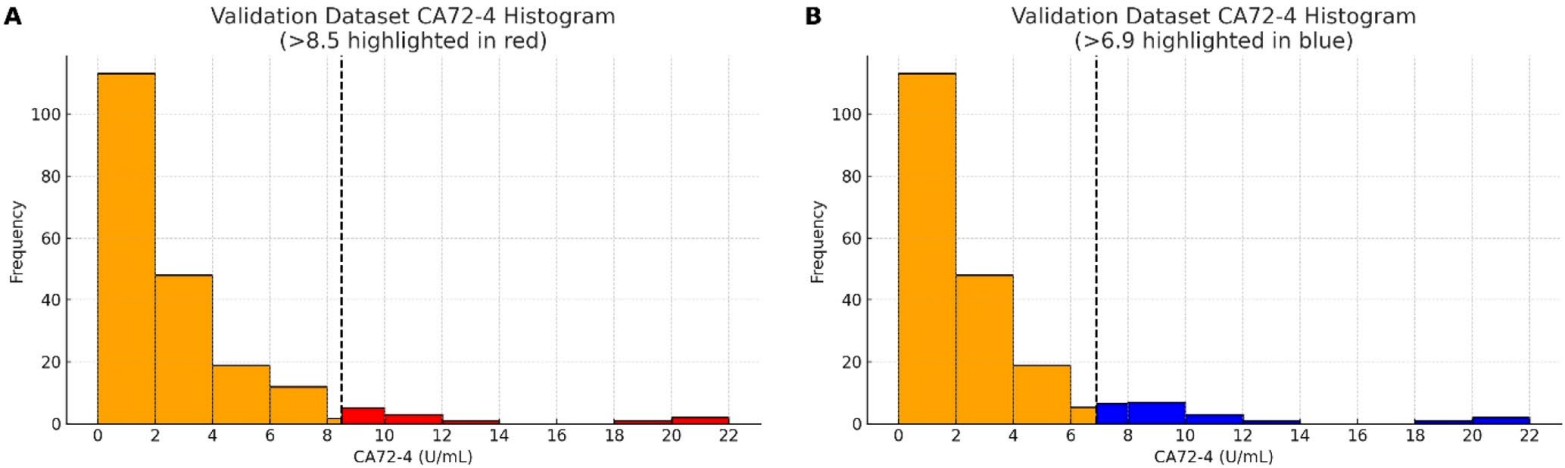
Comparison of the proportion of individuals with elevated CA72‐4 in the validation cohort (*n* = 206) using different reference limits. (A) Using the newly established local reference upper limit of 8.5 U/mL, 4.85% of healthy individuals would be classified as having high CA72‐4. (B) Using the assay manufacturer's reference upper limit of 6.9 U/mL, 8.74% of the same cohort would be labeled as high. Adopting the local reference limit thus substantially reduces false‐positive results in this population.

We applied the newly established reference interval to the validation cohort of 206 healthy individuals. The distribution of CA72‐4 in this validation set by sex and age is provided in Table 5. Using 8.5 U/mL as the upper reference limit, 10 out of 206 individuals (4.85%) in the validation set had CA72‐4 values above this range. This outcome meets our predefined criterion, as approximately 5% of a healthy population would be expected to fall outside the 95% reference interval. Figure 4A illustrates the proportion of validation subjects with elevated CA72‐4 using the 8.5 U/mL cutoff. In contrast, if we applied the assay manufacturer's recommended upper limit of 6.9 U/mL to the validation set, 18 individuals (8.74%) would be labeled as having high CA72‐4 levels (Figure 4B). The exceedance rate with the manufacturer's cut‐off (about 8.7%) is considerably higher than the desirable 5%, indicating that the manufacturer's reference value is not well‐suited to our local population. These results confirm that our locally established reference interval (upper limit 8.5 U/mL) is more appropriate for this population, effectively reducing the false‐positive rate compared to the manufacturer's criteria.

**TABLE 5:** CA72‐4 levels in the healthy validation cohort (Shenzhen, April 2025).

Sex	Age group (years)	*N*	Median (U/mL)
Female	18–40	55	1.33
Female	41–60	40	1.51
Female	61–85	6	2.11
Male	18–40	31	1.96
Male	41–60	62	2.42
Male	61–85	12	2.29

## 4 Discussion

In this study, we successfully established a reference interval for CA72‐4 in a large cohort of healthy adults from Shenzhen and verified its suitability through an independent validation. The upper reference limit was determined to be 8.5 U/mL, which is markedly higher than the 6.9 U/mL provided by the assay manufacturer. This difference is clinically meaningful: using the manufacturer's lower reference limit would have misclassified approximately 8.7% of healthy individuals in our cohort as abnormal, whereas using our local reference limit reduces that to about 4.85%. By adopting the higher local threshold, we can substantially decrease the number of false‐positive results in health screenings. This not only prevents unnecessary alarm for individuals who are in fact healthy, but also avoids needless follow‐up tests and interventions, conserving medical resources and reducing the risk of overdiagnosis and overtreatment. It should be emphasized that a reference interval defines the expected range in healthy people and is not equivalent to a disease diagnostic cutoff. For tumor markers like CA72‐4, clinicians often use higher decision thresholds or consider dynamic changes and combinations of markers when making diagnostic or monitoring decisions [2]. The reference interval, however, plays a crucial role in general health check‐ups and initial assessments: it provides a baseline context to identify results that warrant further evaluation. Our study's establishment of a region‐specific reference interval for CA72‐4 addresses the lack of local baseline data in Shenzhen and ensures that test results are interpreted against an appropriate standard for this population. In future health examination reports in this region, 8.5 U/mL can be used as the reference upper limit for CA72‐4, improving the accuracy of result interpretation. Given that our established upper limit (8.5 U/mL) is higher than the manufacturer's limit (6.9 U/mL), clinicians should exercise caution when interpreting “intermediate” values falling between these two thresholds. In asymptomatic individuals, especially the elderly, mild elevations may reflect benign physiological fluctuations or chronic inflammation rather than malignancy. We recommend that results in this range be interpreted in the context of the patient's full clinical picture.

Our findings show both consistencies and discrepancies with reports from other regions. The absence of a significant sex difference in CA72‐4 levels and the decision not to partition by sex or age is consistent with some studies. For instance, a reference interval study in Wuhan, China (using the Abbott Architect assay), also found no need for sex‐specific partitioning [8]. We observed a slight tendency for CA72‐4 levels to increase with age, particularly in individuals over 60 years, although this did not warrant a separate olderage reference range in our analysis. This trend is biologically plausible, as the elderly may have more subclinical conditions or chronic inflammatory processes that could elevate certain biomarkers. Indeed, a recent study focusing on an elderly population in Southwest China underscored the need for appropriate reference ranges for older adults [9]. Conversely, one study from Beijing reported that CA72‐4 levels actually decreased with age in females, opposite to our observation [6]. The variation in CA72‐4 reference intervals across different regions warrants attention. Our upper limit (8.5 U/mL) is lower than that reported in a Beijing study (18.0 U/mL for females < 60 years) but differs from the manufacturer's claim. These discrepancies may be attributed to regional differences in genetic background, dietary habits, and environmental factors. For instance, the prevalence of *Helicobacter pylori* infection or non‐malignant gastric conditions, which can influence baseline CA72‐4 levels, varies significantly across China. This underscores the necessity of establishing region‐specific RIs rather than relying on a universal standard. Additionally, CA72‐4 can be elevated in some non‐malignant conditions—for example, it was found to be higher in patients with gout, correlating with disease flares [10]. Such factors could contribute to higher baseline CA72‐4 levels in older adults. Clinicians should thus interpret CA72‐4 results in elderly patients with caution, taking into account their overall clinical context to avoid misattributing benign elevations to malignancy. In line with this, a large multicenter study demonstrated that CA72‐4 alone provides limited value in cancer screening of healthy populations [11]. It is important to acknowledge that reference intervals are methodology‐dependent. Our results were obtained using the Roche Cobas e602 system and should not be directly applied to other platforms (e.g., Abbott, Beckman, Mindray) without validation due to differences in antibody specificity and calibration. Furthermore, while establishing a robust RI for CA72‐4 is essential, we agree with current guidelines that CA72‐4 has limited sensitivity as a standalone screening tool. Its clinical value is significantly enhanced when combined with other tumor markers such as CEA and CA19‐9. Our study provides a valuable reference for the Shenzhen population, but it is not without limitations. The cohort was drawn from a single center and predominantly reflects the health screening population of an urban Chinese city; thus, extrapolation to other regions or ethnic groups should be done with caution. Nonetheless, our methodology was rigorous—following international recommendations and using a relatively large sample size—and we included an external validation step to ensure the reference interval's practical applicability. Future research could build on this work by incorporating multi‐center data or by periodically re‐evaluating the reference interval as more local data become available. Additionally, as analytical technologies evolve, reference intervals should be updated to maintain their relevance. Our findings underscore that developing and updating region‐specific reference intervals for tumor markers like CA72‐4 is important for improving the accuracy of laboratory result interpretation, ultimately aiding clinical decision‐making and patient care.

## Funding

This work was supported by Sanming Project of Medicine in Shenzen Municipality (SZSM202311002).

## Data Availability

The data that support the findings of this study are available from the corresponding author upon reasonable request.
